# Population Genetic Analyses and Trichothecene Genotype Profiling of *Fusarium pseudograminearum* Causing Wheat Crown Rot in Henan, China

**DOI:** 10.3390/jof10040240

**Published:** 2024-03-22

**Authors:** Jianzhou Zhang, Jiahui Zhang, Jianhua Wang, Mengyuan Zhang, Chunying Li, Wenyu Wang, Yujuan Suo, Fengping Song

**Affiliations:** 1 Wheat Research Institute, Henan Academy of Agricultural Sciences, Zhengzhou 450002, China; zhangjianzhou00@163.com (J.Z.); jiahuizhang1224@163.com (C.L.); 2 Plant Science College, Tibet Agriculture & Animal Husbandry University, Linzhi 860000, China; lichunying0661@163.com; 3 Institute for Agro-Food Standards and Testing Technology, Ministry of Agriculture, Shanghai Academy of Agricultural Sciences, Shanghai 201403, China; m210300781@st.shou.edu.cn (M.Z.); m230100125@st.shou.edu.cn (W.W.); suoyujuan@saas.sh.cn (Y.S.)

**Keywords:** Fusarium crown rot, *Fusarium pseudograminearum*, trichothecene genotype, mating type

## Abstract

In China, *Fusarium pseudograminearum* has emerged as a major pathogen causing Fusarium crown rot (FCR) and caused significant losses. Studies on the pathogen’s properties, especially its mating type and trichothecene chemotypes, are critical with respect to disease epidemiology and food/feed safety. There are currently few available reports on these issues. This study investigated the species composition, mating type idiomorphs, and trichothecene genotypes of *Fusarium* spp. causing FCR in Henan, China. A significant shift in *F. pseudograminearum-*induced FCR was found in the present study. Of the 144 purified strains, 143 were *F. pseudograminearum*, whereas only 1 *Fusarium graminearum* was identified. Moreover, a significant trichothecene-producing capability of *F. pseudograminearum* strains from Henan was observed in this work. Among the 143 *F*. *pseudograminearum* strains identified, *F. pseudograminearum* with a 15ADON genotype was found to be predominant (133 isolates), accounting for 92.36% of all strains, followed by *F. pseudograminearum* with a 3ADON genotype, whereas only one NIV genotype strain was detected. Overall, a relatively well-balanced 1:1 ratio of the *F. pseudograminearum* population was found in Henan. To the best of our knowledge, this is the first study that has examined the *Fusarium* populations responsible for FCR across the Henan wheat-growing region.

## 1. Introduction

Common wheat is the most important winter crop and provides staple grains for about half of human beings worldwide. The sustained high yields of wheat over the past 40 years have resulted in consistent supply–demand equilibrium [1]. However, on average, about 20% of the global wheat production is lost due to diseases and pests annually [2]. Pathogenic fungi represent a significant constraint to wheat production [1]. Fusarium crown rot (FCR) caused by *Fusarium* species is one such economically important wheat disease. Most recently, Saad et al. [3] examined the impacts of FCR pathogens, *Fusarium pseudograminearum*, and *Fusarium culmorum*, on the wheat root system. The results showed that, in the presence of these pathogen infections, the size and biomass of wheat root were significantly reduced, and significant adverse impacts on the architecture of the root system were also revealed [3], which are all important for yield loss.

According to the surveys conducted to date, FCR is caused by several species of the genus *Fusarium*, with *F. pseudograminearum* being particularly prevalent. The disease has been documented in many other countries throughout the world since its discovery in Queensland, Australia, in 1951, including Africa, America, Europe, and Asia, with the most severe cases occurring in Australia [4,5,6,7,8,9,10]. In Australia, the primary pathogens associated with this disease include *F. pseudograminearum*, *F. culmorum*, and *Fusarium acuminatum* [11]. Similarly, the cultural practices and environmental conditions in the USA and Canada seem to favor the co-occurrence of *F. pseudograminearum* and *F. culmorum* on spring wheat [12,13]. On the other hand, *Fusarium avenaceum*, *F. culmorum*, and *F. graminearum* are the three well-known species associated with FCR in wheat in Spain, and *F. pseudograminearum* was first found in a commercial field located in Córdoba, Spain, in 2016 and formally reported in 2018 [14].

Worldwide, severe damage to wheat production caused by FCR has been reported during the past two decades. The FCR-induced crop losses in Australia have indicated that the disease can cause an average annual reduction of about 10% in wheat grain yield under natural inoculum levels, which is estimated to be AUD 88 million [15]. On the other hand, the prevalence and epidemic of this disease are largely influenced by climate factors. As reviewed by Scherm et al. [16], drought conditions increase the susceptibility of the plant rather than the virulence of the fungus. Thus, the disease is promoted by hot and dry weather at crop anthesis and maturation [17], and more severe yield losses have been reported in semi-arid wheat-growing regions around the world [11]. In the Pacific Northwest of the USA, FCR caused by *F. pseudograminearum* is estimated to routinely cause up to 35% yield loss in wheat grain [18]. 

*Fusarium*-induced crown rot of wheat is not a new disease in China, while *F. pseudograminearum* has emerged as a major pathogen causing FCR and poses a serious threat to wheat production. FCR in wheat caused by *F. pseudograminearum* was first reported by Li et al. [19] in Henan, China, in 2011. The species was subsequently documented in other geographic regions in China, such as Jiangsu [20] and Hebei provinces [21], associated with FCR, along with Fusarium head blight (FHB) of wheat [22], and, occasionally, it was found in other plant hosts [23,24]. Currently, *F. pseudograminearum*-induced FCR has become more serious in the main wheat-growing region in China and is most severe in Henan province, which has the largest wheat-growing areas and yields in China. The disease has been listed as one of the four major wheat diseases by the Chinese government. 

As a member of the genus *Fusarium*, in addition to causing yield losses, *F. pseudograminearum* is capable of producing various *Fusarium* toxins, such as trichothecenes represented as deoxynivalenol (DON) and zearalenone. Grain and straw tissue with high mycotoxin levels cannot be used for food and feed products. Many countries defined strict limits for several mycotoxins, including DON and zearalenone, regarding the commercialization of unprocessed kernels and the food products obtained from different cereals [25]. Of the 44 samples collected from different field sites in Queensland and New South Wales in 2010, DON concentrations in straw from 26 sites exceeded 1 mg/kg, with a percentage of 59% [17]. Similarly to the situation in the *Fusarium graminearum* species complex (FGSC), the main pathogen of wheat FHB worldwide, three different trichothecene genotypes were identified in *F. pseudograminearum* species [6,20,26,27], namely, 3-acetyl DON, 15-acetyl DON, and nivalenol (NIV) types. The different toxicological effects observed among the three trichothecene-producing strains make their identification in a given area important in terms of food/feed safety and disease management. Moreover, some mycotoxins produced by *Fusarium* strains can function as virulence factors during the pathogen infection process, enhancing pathogen virulence or aggressiveness in certain host plants [28,29].

Although several surveys about pathogenic fungi *F. pseudograminearum* and their induction of FCR have been conducted in Henan, little is known about the *F. pseudograminearum*’s population genetic structure and distribution, or the prevalence of their trichothecene genotype diversity in wheat in Henan. Xu et al. [30] surveyed the spatial distribution of pathogenic fungi associated with the crown rot of wheat in Henan, and their relationship with climate variables was also discussed. Similarly, Zhou et al. [31] investigated the distribution of the pathogens associated with FCR in the Huanghuai wheat-growing region (including Henan) and examined the pathogenic and genetic diversity within and among the predominant species. However, no information was provided about the mating type idiomorph and toxin potential of *F. pseudograminearum* populations in all these investigations. Therefore, this study is focused on the mating type idiomorph and mycotoxin genotype distribution of the *F. pseudograminearum* population in Henan. 

## 2. Materials and Methods

### 2.1. Sample Collection 

**Figure 1:**
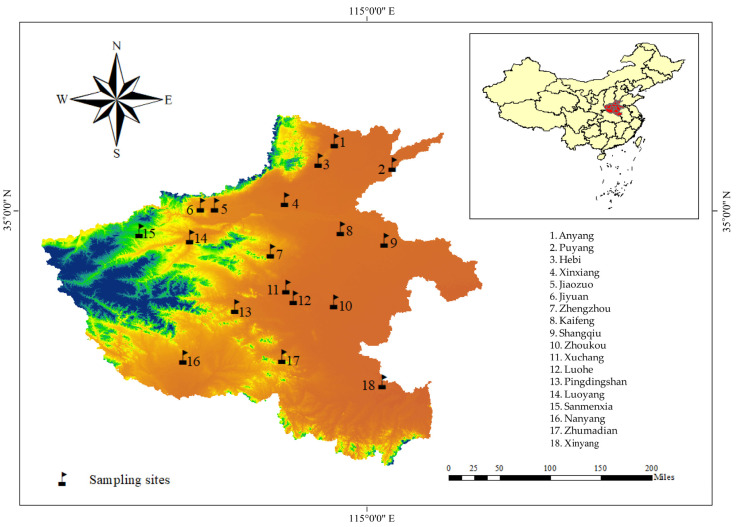
Locations of sampling sites in the 18 cities of Henan (indicated with flags), China, in 2023.

Whole wheat straws exhibiting typical symptoms of crown rot from winter wheat fields in the main wheat-growing regions of Henan were collected in 2023 prior to harvest. Wheat samples were collected from 18 locations across the main wheat-growing regions in Henan province (Figure 1). At each of these sites, wheat samples were collected from commercial fields following a previously described strategy [32]. For each site, 10 to 15 whole plants were collected with a minimum distance of 500 m. The samples were collected by uprooting whole plants, including the roots. Geographic data, including the latitude and longitude of individual sampling sites, were recorded with the aid of a Global Positioning System (Kubota, T16). The average temperature, average relative humidity, and total precipitation of different sampling sites for April, May, and June were obtained from the meteorological department (Table 1).

### 2.2. Fungal Isolation and Culture

Fungal culture isolation was performed as previously described by Khudhair et al. [6], with minor changes. In brief, the symptomatic crown/sub-crown tissues were cut into small segments (about 2 cm in length). The segments were then surface-sterilized (1% sodium hypochlorite, 3 min), rinsed twice with sterile distilled water, transferred onto sterile filter paper, and dried in a biosafety cabinet. The dried sections were placed on potato dextrose agar (PDA) medium supplemented with streptomycin (working solution concentration 0.1 g/L). For each wheat plant, one section was selected for fungal isolation. After 3–5 days of incubation in a constant-temperature incubator at 25 °C, fungal colonies recovered from the sections were subsequently transferred into fresh PDA plates with sterile toothpicks. The resulting fungal colony was subcultured several times on PDA until a pure culture was obtained for individual strains. The single-spore isolation of *Fusarium*-like colonies was performed as described by Zhang et al. [33]. The resulting fungal strains were routinely grown on PDA plates at 25 °C. For short-term preservation, all strains were grown on PDA stock tubes at 4 °C.

### 2.3. Genomic DNA Extraction

Fungal strains were inoculated onto fresh PDA plates, and then cultured at 25 °C for 3 days in the dark for mycelia induction. Aerial mycelia were harvested using sterile toothpicks and transferred into 2 mL microcentrifuge tubes containing 650 mL CTAB buffer. A similar protocol, previously published by Wang et al. [34], was followed for DNA extraction, except that a water bath approach (65 °C, 40 min) was added after the homogenization of fungal mycelia. The resultant DNA was suspended in sterile deionized water, quantified with the aid of a spectrophotometer. Partial DNA was selected and diluted into a working concentration (about 20 ng/μL) with water without nuclease. All DNA samples were kept frozen at −20 °C until required for use.

### 2.4. Fusarium pseudograminearum Confirmation by Specific Primers

All obtained *Fusarium* strains were initially identified according to the morphological and cultural characteristics, and then confirmed using a molecular assay. To confirm that all 144 purified strains from Henan province were correctly identified as *F. pseudograminearum*, fungal DNA was subjected to PCR amplification using species-specific primer sets Fp1-1 and Fp1-2 (Table 2) [35]. The PCR amplifications were carried out in a 20 μL volume reaction mixture containing 2 μL 10 × EasyTaq PCR buffer (Transgen Biotech, Beijing, China), 1 μL of template DNA (20 ng), 0.2 μL of each primer (10 μM), and 16.6 μL sterile water. The amplifications were conducted in a T100 Thermal Cycler (Bio-Rad, Hercules, CA, USA) using the following program: initial denaturation at 94 °C for 4 min, followed by 30 cycles of 30 s at 94 °C, 30 s at 60 °C, and 30 s 72 °C, with a final extension at 72 °C for 5 min. Amplicons were separated using agarose gel (1.5%) electrophoresis in TEA buffer, stained with nucleic acid fluorescent stain reagent (Transgen Biotech, Beijing, China), and photographed under UV light. An expected specific fragment of 520 bp would be amplified from the *F. pseudograminearum* strains. The strains that failed to amplify any fragment with primers Fp1-1 and Fp1-2 were further amplified using primer pair Fg16F/R (Table 2) [36] to identify the species for FGSC. The primers used in this study are listed in Table 2.

### 2.5. Trichothecene Genotype Determination of Fusarium pseudograminearum

A published PCR assay for the trichothecene genotyping determination of FGSC was used to analyze the only FGSC strain [34]. To determine the trichothecene genotype of each *F. pseudograminearum* strain, a recently reported trichothecene genotype-specific assay was performed, as described by Deng et al. [37], targeting *Tri8* (an essential gene encoding a deacetylase) within the core trichothecene biosynthetic gene cluster. All of the obtained DNA samples were analyzed using three primer sets of 3AT8-1/3AT8-2, 15AT8-1/15AT8-2, and NIVT8-1/NIVT8-2 (Table 2) for 3ADON, 15ADON, and NIV genotype strains, respectively. PCR amplifications were performed in a total reaction volume of 20 μL, as described in Deng et al. [37]. The PCR assay was run in a Bio-Rad T100 thermal cycler using the following program: 2 min initial denaturation at 94 °C, followed by 30 cycles consisting of 30 s at 94 °C, 30 s at 60 °C, and 50 s at 72 °C, with a final elongation at 72 °C for 5 min, and a refrigeration step at 12 °C. Amplicons were separated in 1.2% agarose gels run in a TAE buffer. For these three primer sets, the fragments amplified from 3ADON, 15ADON, and NIV genotype strains are expected to be 424 bp, 827 bp, and 397 bp, respectively, in length.

### 2.6. Mating Type Idiomorph Determination of Fusarium pseudograminearum

A previously reported PCR approach to the mating-type idiomorph assay was applied for the screening of all *F. pseudograminearum* strains [38]. Two mating-type primer pairs, fusALPHAfor/rev (expected fragment of 200 bp in size, specific to *MAT-1* strains) and fusHMGfor/rev (expected fragment of 260 bp in size, specific to *MAT-2* strains) (Table 2), were used for *MAT-1* and *MAT-2* idiomorphs, respectively. The same 20 μL of the reaction system was used for the amplification of these two mating-type markers, which contained 2 μL 10 × EasyTaq PCR buffer (Transgen Biotech, Beijing, China), 0.2 μL of each primer (10 μM), 1 μL of template DNA (20 ng), and up to 20 μL of sterile water. The thermal cycler program comprised an initial denaturation step at 94 °C for 4 min; followed by 30 cycles with 94 °C for 30 s, 30 s at 60 °C for primer pair fusALPHAfor/rev, or 62 °C for primer pair fusHMGfor/rev, and 72 °C for 20 s; and a 5 min final extension at 72 °C. The amplified DNA fragments were separated using gel electrophoresis in 1.5% agarose gels, stained, and visualized as mentioned above. The mating type ration of each site was analyzed using a chi-square test against an expected ration of 1:1 at a significance level of *p* = 0.05.

## 3. Results

### 3.1. Pathogen Identification

In the current study, a total of 144 *Fusarium* strains were obtained from FCR symptomatic wheat plants collected from 18 sites in the wheat-growing regions of Henan from commercial fields in 2023. According to the species-specific PCR assay with primer pair Fp-1/2, 143 strains produced a 520 bp fragment, indicative of *F. pseudograminearum*, accounting for 99.31% of all the obtained strains. The remaining strain, isolated from Zhoukou, was subsequently subjected to PCR assays with primer pair Fg16F/R, a species-specific marker for FGSC strains. The results demonstrated that a 410 bp product was obtained from this strain with the primer set Fg16F/R, indicating that it is FGSC. Thus, combined with morphological and molecular techniques, 1 strain of FGSC and 143 of *F. pseudograminearum* were identified.

### 3.2. Trichothecene Genotype Determination

The PCR assays of the only FGSC strain with the primer pair Tri13P1/2 indicated that the strain was typed as a 15ADON genotype. The *Tri8*-based genotype PCR results revealed the presence of three different patterns of PCR products, representing 3ADON, 15ADON, and NIV genotypes. Of the 143 *F. pseudograminearum* strains that were assayed, 133 (93.01%) were determined as the 15ADON genotype, and 9 (6.29%) as the 3ADON genotype, while only 1 NIV genotype strain was identified (account for 0.70%) (Table 3). The *F. pseudograminearum* toxin potential assay results indicated that the 15ADON genotype significantly outnumbered the 3ADON genotype. Thus, 15ADON is the predominant mycotoxin produced by the *F. pseudograminearum* strains in Henan. Overall, *F. pseudograminearum* with a 15ADON genotype was found to be predominant (133 isolates), accounting for 92.36% of all 144 detected strains.

### 3.3. Mating Type Determination

We know that FGSC is homothallic, whereas *F. pseudograminearum* is presumed to be a heterothallic pathogen, and individuals only have one of the two mating-type idiomorphs in their genomes. Thus, except for the 1 FGSC strain, all 143 *F. pseudograminearum* strains were analyzed for mating type.

The PCR assay results with the primer sets fusALPHAfor/rev and fusHMGfor/rev showed that each of the *F. pseudograminearum* strain amplified a single mating type, idiomorph-specific band, either with fusALPHAfor/rev (*MAT-1*) or fusHMGfor/rev (*MAT-2*). Both mating types were identified in the *F. pseudograminearum* population of Henan. The results revealed that, of the 143 *F. pseudograminearum* strains, 80 strains (55.9%) and 63 strains (44.1%) had *MAT-1* and *MAT-2* mating types, respectively (Table 4). Considering the 143 *F. pseudograminearum* strains as a whole, the mating type ratio was not significantly different from 1:1 (*p* = 0.05).

However, as shown in Table 4, significant deviations from the expected ratio of 1:1 were found for the two mating types within several sampling sites. For example, the Puyang, Xuchang, and Zhumadian populations had a significantly higher proportion of *MAT-1*, while a much higher proportion of *MAT-2* was found in Anyang compared to *MAT-1*. The χ^2^ test indicated that the 1:1 mating type ratio could not be rejected at a 95% confidence level for the two mating types in Hebi, Xinxiang, Jiaozuo, Jiyuan, Zhengzhou, Kaifeng, Shangqiu, Zhoukou, Luohe, Pingdingshan, Luoyang, Sanmenxia, Nanyang, and Xinyang (Table 4).

## 4. Discussion

Wheat production in China and worldwide continuously faces challenges from severe fungal diseases, such as FHB and FCR, caused by fungi belonging to the genus *Fusarium*. Wheat crown rot occurred relatively late in China, but in recent years, it has expanded dramatically for unknown reasons. A good example is Henan. In China, Henan province has the largest wheat-growing areas and yields. According to national statistical data, in recent years, the proportion of wheat-planting area in Henan province is around 20% of the country, with a stable wheat-planting area of over 56,853 square kilometers throughout the year, and wheat production accounting for one-fourth of the whole country. Comprehensive studies have been conducted on FHB worldwide during the past two decades [39]. Tremendous progress has been made in understanding the population structure, trichothecene genotype and biosynthesis, genetic diversity, etc., of the *Fusarium* pathogens causing FHB. However, until now, relatively few studies have been reported about the *F. pseudograminearum* populations causing FCR with respect to these issues, especially in China. The present work aimed to characterize the distribution of mating type idiomorphs and trichothecene genotypes of the *F. pseudograminearum* population in Henan.

In the 2023 growing season, severe FCR caused by *F. pseudograminearum* occurred in Henan due to the hot and dry weather that occurred from April to May. The estimated yield losses were as high as 30% in some fields. The current study investigated the pathogen species, mating type, and toxin potential of FCR-causing *Fusarium* strains collected from the 18 cities of Henan in 2023. All of the isolated strains belonged to the *Fusarium* genus. Among all these strains, it is obvious that *F. pseudograminearum*, representing a predominant population of FCR in wheat, accounts for 99.31%, while FGSC is rare (less than 0.70%). According to the newest prediction by the national agricultural technology center, the estimated occurrence area of FCR in China is about 40,000 square kilometers in the coming 2024 growing season. These figures clearly show that, at present, FCR is an economically important wheat disease in Henan.

A previous study by Deng et al. [40] has described the mating-type idiomorphs of *F. pseudograminearum* strains from eastern China. In Jiangsu, the ratios of the two mating types of the *F. pseudograminearum* strains deviated significantly from an expected 1:1 ratio, while a relatively more balanced mating type ratio was detected in Shandong [40]. In this study, for the first time, we determined the mating types of *F. pseudograminearum* strains in Henan. Our results revealed that *F. pseudograminearum* strains have both mating idiomorphs, which is consistent with previous studies indicating that the pathogen segregates for both *MAT-1* and *MAT-2* [4,6,7,40,41]. Although significant ratio differences were detected for the two mating types, *MAT-1* and *MAT-2*, between individual sampling sites, a well-balanced 1:1 ratio of the *F. pseudograminearum* population in Henan was found overall. On the other hand, our findings indicated that, to some extent, *F. pseudograminearum* in China originated in Henan. In addition, Henan shares a border with Shandong, both of which belong to the North China Plain; hence, they might be regarded as a single agroecological area. The consistence in mating type composition between Henan and Shandong may thus imply that the two provinces have a comparable risk of being invaded by this pathogen, lending weight to the theory that *F. pseudograminearum* originated in north-central China [40].

In addition to losses in yield and seed quality, food or feed safety is by far the greatest concern, as mycotoxin contamination was introduced by *Fusarium* species. The genotype structure of the *F. pseudograminearum* population in a certain area can be useful for a risk assessment of the potential impacts on mycotoxin [42]. In the present study, a high prevalence (93.01%) of the *F. pseudograminearum* strain with the 15ADON genotype was identified in Henan. This finding is in line with observations in other parts of China (Shandong), where 15ADON is the predominant population [40]. Within the *F. pseudograminearum* strains, the 3ADON and NIV populations are quite infrequent (6.29% and 0.70%, respectively) in Henan, in contrast to the situation previously reported in Jiangsu [40]. The low frequencies of 3ADON and NIV populations in Henan wheat may suggest a limited impact of 3ADON and NIV in Henan crops, but requires constant monitoring, as a chemotype shift may occur over time [43,44,45]. However, studies conducted in Australia demonstrated that more than 90% of the *F. pseudograminearum* strains showed the 3ADON genotype, with the 15ADON genotype occasionally detected at a low frequency [6,7]. According to the results by Ward et al. [43,46], *Fusarium* trichothecene chemotype polymorphism was maintained by multiple speciation events through adaptive evolution, indicating that their chemotype differences may have a significant impact on pathogen fitness [43,46,47,48,49]. It is well known that dynamic changes in species structure and genotype proportion have been observed in the wheat scab pathogen FGSC worldwide [43,45,50]. However, it is still unclear whether similar phenomena will occur in *F. pseudograminearum* in the future, and continuous monitoring of the pathogen population is required.

Drastic changes occurred in the species composition causing FCR in wheat in Henan in the past two decades. A previous study by Zhang et al. [20] was conducted to explore the *Fusarium* species causing wheat crown rot in the five major winter wheat-growing provinces (Anhui, Hebei, Henan, Jiangsu, and Shandong) from 2009 to 2013. The results showed that, of the 25 Henan strains, 17 were *F. asiaticum*, 7 were *F. graminearum*, and the remaining 1 was identified as *F. acuminatum*. Of particular note, the only *F. pseudograminearum* strain that was obtained was isolated from Jiangsu, while this pathogen was absent in Henan in their survey [20]. The results by Zhang et al. [20] clearly showed that *F. asiaticum* was the main causal agent of wheat crown rot in China, followed by *F. graminearum*. This is in contrast with our results, which show that *F. pseudograminearum* acts as the main species associated with FCR in Henan. Such dynamic changes in the species composition of wheat scab pathogen have been well documented worldwide, which may result in natural selection and adaptive evolution in *Fusarium* populations [43,45,50]. Moreover, according to the results from a range of analytical tools, Chakraborty et al. [51] concluded that, generally, *F. pseudograminearum* is more aggressive than *F. graminearum*. This competitive advantage in pathogenicity will inevitably lead to the replacement of *F. graminearum* by *F. pseudograminearum*.

Since its first detection in Henan in 2011, with a frequency of 5.97% [19], *F. pseudograminearum*-induced FCR has become more and more severe in regions of China, with the most severe occurring in Henan, posing a serious threat to wheat production [20]. The distribution frequency of the pathogenic fungi associated with root and crown rot of winter wheat in the North China Plain (including Henan and its neighboring cities of Handan in Hebei and Heze in Shandong) was determined from 2013 to 2016 by Xu et al. [30]. The results showed that *F. pseudograminearum* was the predominant pathogen recovered from wheat root and stem samples, with isolation frequencies of 14.9% and 27.8%, respectively [30]. Likewise, Zhou et al. [31] investigated the distribution and diversity of the pathogens associated with FCR in the Huanghuai wheat-growing region (Henan, Shandong, Hebei, Shanxi, Shaanxi, Jiangsu, and Anhui) in China from 2013 to 2016. Zhou et al. [31] isolated a large number of *Fusarium* strains from Henan, totaling 528, with 313 strains identified as *F. pseudograminearum*. Their results indicated that the isolation frequency of *F. pseudograminearum* from Henan was 59% [31]. In the present study, a high percentage of *F. pseudograminearum* (99.31%) was identified in Henan, which is consistent with findings by Zhou et al. [31] and Xu et al. [30]. Conversely, we recovered only one strain of *F. graminearum* associated with wheat FCR in Henan. Our data are in accordance with the results of Zhang et al. [20], Xu et al. [30], and Zhou et al. [31], showing that *Fusarium* strains from FGSC are also the causal agents of FCR in Henan, and the pathogens co-exist with *F. pseudograminearum* in the field, even at low frequency levels. A similar surveillance of *Fusarium* wheat crown rot pathogens outside Henan was conducted in Jiangsu and Shandong provinces from 2014 to 2016 in China [40]. Of the 617 *Fusarium* isolates, 372 (60.3%) were identified as *F. pseudograminearum* [40], which is closer to the results obtained in our study.

According to the previous surveys and the results of the present study, *F. pseudograminearum* is becoming a predominant causative pathogen of crown rot of wheat in China. The frequency of *F. pseudograminearum* increased more than 16-fold between 2011 (5.97%) and 2023 (99.31%) in Henan wheat-growing regions. Furthermore, the current increase in FCR incidence and severity in Henan is most likely due to the substantial invasion of *F. pseudograminearum* populations, and a clear dramatic cline in *F. pseudograminearum* species can be seen over time. In this study, the much higher frequency of *F. pseudograminearum* suggests that this pathogen had already become a dominant population in Henan province before this survey.

The recent increase in the incidence and severity of FCR in Henan, and even in China, resulted in many complicating factors. Except for the fast invasion or high frequency of *F. pseudograminearum* observed in this study, this probably could be explained, at least in part, as follows. First, as reviewed by Scherm et al. [16], climatic conditions play key roles in increasing the prevalence of *Fusarium* species, which can drastically reverse the previous species distribution. Henan province is located in central eastern China, largely in the warm temperate zone, with the southern section extending into the subtropical zone. This region has a continental monsoon climate, which moves from the northern subtropical to the warm temperate zone. The climate conditions are suitable for the *F. pseudograminearum* population and may induce disease [30]. A similar correlation to the Henan results was previously reported in the arid zones of the Eastern Australia (Queensland and New South Wales) [52] and the Pacific Northwest of the USA [53,54]. The climatic conditions of the plant sampling sites in our current study are provided in Table 1. Due to the one-year data not being sufficient to discuss the influence of climatic conditions on disease occurrence, no further discussions are made here. However, long-term comprehensive studies should be carried out to reveal their contributions to the disease epidemic with respect to different climatic factors. Second, the previous crop and residue management are key factors in the development of FCR [16]. In order to increase the content of soil organic matter in farmland ecosystems, straw returning is considered an effective form of field management to increase soil fertility and reduce the use of chemical fertilizers, which has been widely used in China in recent years. The agronomic practice, as reported by Backhouse [55] and Deng et al. [40], may increase the preliminary infection source and promote the occurrence of wheat crown rot in Henan, or even more widely in China. Wheat-maize rotation, a very common planting practice in Henan, will also boost the inoculums and, consequently, the chances of increasing FCR severity [16]. As recently reported by Knight and Sutherland [56], Župunski et al. [57], Boamah et al. [58], and Li et al. [59], the impacts of wheat varieties, *Fusarium* species, and even their potential trichothecene genotypes on disease epidemics cannot be ignored. Third, *F. pseudograminearum* is heterothallic, and abundant genetic diversity was found in the Henan *F. pseudograminearum* population [31], which provides a force driving the evolution of the pathogen populations and may play an essential role in disease epidemiology [60]. Fourth, the confirmed diagnosis and isolation of *F. pseudograminearum* from wheat and other plant hosts clearly indicates a broad host spectrum for the pathogen [21,22,23,24,61], which is also essential for the pathogen’s survival and its ability to adapt to changing environments.

## 5. Conclusions

In the present study, *F. pseudograminearum* with a 15ADON genotype was found to be the predominant population for FCR in wheat in Henan. The results show that, overall, the *F. pseudograminearum* population segregates for *MAT-1* and *MAT-2* idiomorphs with a 1:1 expected mating type ratio. This is the first survey to identify the *F. pseudograminearum* mating type and trichothecene genotype in the main wheat-growing regions of Henan. The findings provided here address a significant research issue regarding *F. pseudograminearum* population genetic analysis and provide valuable data for FCR control. Furthermore, the high frequency of the 15ADON genotype *F. pseudograminearum* strains also highlighted that much more attention should be paid to the pathogen with respect to food/feed safety issues. Further work is required to obtain a better understanding of the spatial and temporal dynamics, genetic diversity, and management of this pathogen.

## Figures and Tables

**Table 1:** Climatic conditions of different sampling sites in Henan from April to June 2023.

Sample Site	April			May			June		
	Mean Temperature (°C)	Mean Relative Humidity (%)	Precipitation (mm)	Mean Temperature (°C)	Mean Relative Humidity (%)	Precipitation (mm)	Mean Temperature (°C)	Mean Relative Humidity (%)	Precipitation (mm)
Anyang	15.6	59.0	118.2	20.5	67.0	133.4	27.0	52.0	26.6
Puyang	15.5	65.0	194.0	20.5	66.0	24.2	27.5	62.0	35.7
Hebi	15.3	65.0	91.0	20.3	70.0	77.3	27.3	53.0	56.0
Xinxiang	15.8	65.0	76.2	20.8	70.0	95.4	27.4	57.0	11.3
Jiaozuo	15.6	62.0	115.9	21.5	67.0	45.9	27.4	57.0	98.5
Jiyuan	15.5	65.0	56.3	20.3	71.0	194.3	26.7	58.0	60.9
Zhengzhou	16.5	63.0	29.9	21.5	75.0	174.2	27.5	68.0	44.3
Kaifeng	16.5	61.0	65.8	21.3	67.0	119.0	27.3	60.0	31.7
Shangqiu	15.4	68.0	77.7	20.3	73.0	133.9	26.1	66.0	63.2
Zhoukou	16.3	67.0	63.2	20.9	73.0	140.9	26.6	67.0	81.2
Xuchang	15.5	69.0	54.0	20.5	73.0	131.0	26.3	66.0	57.2
Luohe	16.3	60.0	166.5	21.5	72.0	111.7	25.5	66.0	53.7
Pingdingshan	15.4	58.0	102.7	20.5	68.0	35.7	26.3	65.0	13.7
Luoyang	15.6	59.0	84.8	20.1	69.0	123.9	25.6	62.0	51.2
Sanmenxia	15.1	59.0	47.4	18.5	68.0	104.9	25.5	58.0	8.8
Nanyang	16.6	67.0	42.7	20.7	73.0	122.1	25.0	72.0	194.1
Zhumadian	16.4	68.0	102.2	20.6	76.0	177.1	25.7	70.0	213.0
Xinyang	16.5	67.0	39.5	20.5	76.0	143.2	27.5	71.0	85.5

**Table 2:** Primers used in this study.

Primer	Nucleotide Sequence (5′ to 3′)	Reference
Tri13P1	CTC(G/C)ACCGCATCGAAGA(G/C)TCTC	[34]
Tri13P2	GAA(G/C)GTCGCA(A/G)GACCTTGTTTC	[34]
Fp1-1	CGGGGTAGTTTCACATTTC(C/T)G	[35]
Fp1-2	GAGAATGTGATGA(C/G)GACAATA	[35]
Fg16F	CTCCGGATATGTTGCGTCAA	[36]
Fg16R	GGTAGGTATCCGACATGGCAA	[36]
3AT8-1	CCTTATGACTCCCCCGATGTCG	[37]
3AT8-2	TGTTTACCACCAGACCGGAC	[37]
15AT8-1	AAGCGCGCTCATGTCAGTCCAAGTT	[37]
15AT8-2	GCCCACCGACAGTATTCCTT	[37]
NIVT8-1	GTACACCGCGAGCGCTATTTCTTCT	[37]
NIVT8-2	CGTGAGACCCAACAGCAT	[37]
fusALPHAfor	CGCCCTCT(G/T)AA(C/T)G(C/G)CTTCATG	[38]
fusALPHArev	GGA(A/G)TA(A/G)AC(C/T)TTAGCAAT(C/T)AGGGC	[38]
fusHMGfor	CGACCTCCCAA(C/T)GC(C/T)TACAT	[38]
fusHMGrev	TGGGCGGTACTGGTA(A/G)TC(A/G)GG	[38]

**Table 3:** Trichothecene genotypes of *Fusarium pseudograminearum* strains isolated from Henan, China, in 2023.

City	Sampling Site	Strain Number	Number of Strains		
			3ADON	15ADON	NIV
Anyang	35°59′42.702″ N, 114°32′54.676″ E	8	0	8	0
Puyang	35°39′27.836″ N, 115°22′20.830″ E	8	1	7	0
Hebi	35°42′51.059″ N, 114°19′8.590″ E	7	0	7	0
Xinxiang	35°9′28.497″ N, 113°50′0.940″ E	5	1	4	0
Jiaozuo	35°5′5.527″ N, 112°49′58.265″ E	6	1	5	0
Jiyuan	35°5′10.142″ N, 112°38′18.015″ E	11	2	9	0
Zhengzhou	34°25′33.189″ N, 113°38′2.691″ E	11	0	11	0
Kaifeng	34°44′36.787″ N, 114°37′56.559″ E	10	1	9	0
Shangqiu	34°34′47.536″ N, 115°15′47.639″ E	7	0	7	0
Zhoukou	33°42′38.148″ N, 114°32′32.892″ E	5	0	5	0
Xuchang	33°54′55.083″ N, 113°51′4.283″ E	14	1	13	0
Luohe	33°46′9.229″ N, 113°57′52.073″ E	7	0	7	0
Pingdingshan	33°38′31.425″ N, 113°7′24.507″ E	9	0	8	1
Luoyang	34°38′4.856″ N, 112°29′5.618″ E	5	0	5	0
Sanmenxia	34°43′16.480″ N, 111°45′25.842″ E	5	0	5	0
Nanyang	32°55′18.865″ N, 112°23′3.635″ E	10	0	10	0
Zhumadian	32°55′33.584″ N, 113°48′8.004″ E	8	2	6	0
Xinyang	32°32′12.907″ N, 115°13′54.549″ E	7	0	7	0
**Total**	**…**	**143**	**9**	**133**	**1**

**Table 4:** Mating type data of *Fusarium pseudograminearum* strains isolated from Henan, China, in 2023.

City	Strain Number	Number and Frequency (%)		χ^2^	*p*-Value
		*MAT-1*	*MAT-2*		
Anyang	8	1 (12.5)	7 (87.5)	4.500	0.034 *
Puyang	8	8 (100.0)	0 (0)	8.000	0.005 *
Hebi	7	5 (71.4)	2 (28.6)	1.286	0.257
Xinxiang	5	2 (40.0)	3 (60.0)	0.200	0.655
Jiaozuo	6	2 (33.3)	4 (66.7)	0.667	0.414
Jiyuan	11	4 (36.4)	7 (63.6)	0.818	0.366
Zhengzhou	11	8 (72.7)	3 (27.3)	2.273	0.132
Kaifeng	10	3 (30.0)	7 (70.0)	1.600	0.206
Shangqiu	7	2 (28.6)	5 (71.4)	1.286	0.257
Zhoukou	5	2 (40.0)	3 (60.0)	0.200	0.655
Xuchang	14	11 (78.6)	3 (21.4)	4.571	0.033 *
Luohe	7	2 (28.6)	5 (71.4)	1.286	0.257
Pingdingshan	9	5 (55.6)	4 (44.4)	0.111	0.739
Luoyang	5	3 (60.0)	2 (40.0)	0.200	0.655
Sanmenxia	5	4 (80.0)	1 (20.0)	1.800	0.180
Nanyang	10	7 (70.0)	3 (30.0)	1.600	0.206
Zhumadian	8	7 (87.5)	1 (12.5)	4.500	0.034 *
Xinyang	7	4 (57.1)	3 (42.9)	0.143	0.705
**Total**	**143**	**80 (55.9)**	**63 (44.1)**	**2.021**	**0.155**

* A mating type ratio significantly different from 1:1 (*p* = 0.05).

## Data Availability

All relevant data generated or analyzed during this study are included in this manuscript.
